# Stimulating Environmental and Health Protection Through Utilizing Statistical Methods for Climate Resilience and Policy Integration

**DOI:** 10.3390/ijerph22030331

**Published:** 2025-02-24

**Authors:** Sanaa Kaddoura, Rafiq Hijazi, Nadia Dahmani, Reem Nassar

**Affiliations:** 1 Departmen of Computing and Applied Technology, College of Technological Innovation, Zayed University, Abu Dhabi P.O. Box 144534, United Arab Emirates; reamnassar@hotmail.com; 2 Department of Mathematics and Statistics, College of Natural and Health Sciences, Zayed University, Abu Dhabi P.O. Box 144534, United Arab Emirates; rafiq.hijazi@zu.ac.ae; 3 Department of Information Systems and Technology Management, College of Technological Innovation, Zayed University, Abu Dhabi P.O. Box 144534, United Arab Emirates; nadia.dahmani@zu.ac.ae

**Keywords:** climate change policy, climate risk perception, extreme weather events, sustainability and adaptation, perceived adaptive capacity

## Abstract

Climate change, a critical global challenge, is evident in rising global temperatures, shifting precipitation trends, and extreme weather events, including floods, heatwaves, and rising sea levels. The impacts of climate change not only endanger physical health but also affect mental well-being, particularly among populations experiencing frequent or severe climate-related events. Understanding individual perceptions of climate risks and adaptive capacities is crucial for developing strategies that promote health resilience and environmental protection. This paper examines how risk perceptions, direct experiences with extreme weather, and perceived adaptive capacities influence climate change protection measures and support for relevant policies. Data were gathered from 291 respondents in the United Arab Emirates using structured questionnaires. The data were analyzed using descriptive statistics, reliability analysis, Cronbach’s alpha, Spearman correlation analysis, and multiple regression analysis to determine key predictors of policy support. The results indicate that age is positively correlated with policy support (*ρ* = 0.16, *p* = 0.001), while gender also plays a role, with women showing greater risk perception and stronger policy support than men. In contrast, formal education and employment status do not significantly impact policy endorsement or climate adaptation behaviors. These findings suggest that awareness-based interventions alone may be insufficient to drive climate action. Instead, policies should leverage older individuals’ experiences, enhance workplace and community-based climate engagement, and prioritize hands-on, action-oriented education to bridge the gap between climate knowledge and adaptive behavior.

## 1. Introduction

Climate change represents a critical global issue characterized by escalating temperatures and an increased frequency of extreme weather events such as floods, droughts, and heatwaves [1]. Climate change has lately been exacerbated by human activities such as urbanization and digitization. Urbanization contributes to the worsening of the climate change crisis through the phenomenon of Urban Heat Islands (UHIs) [2], where densely populated cities experience higher temperatures than surrounding rural areas [3]. About 55% of the world’s population resides in urban areas, projected to exceed 68% by 2050 [4]. This urban expansion increases energy demands, particularly for cooling, and increases pollutant concentrations, ecological footprints, and health risks [5]. Studies reveal that for each degree Celsius increase in temperature, urban areas may see a rise in peak electricity demand from 0.45% to 4.6%, showing how urbanization participates in climate change [6].

The digitization and growth of industrial regions often grapple with emissions that contribute to environmental degradation. The construction and building sectors drive economic growth and development but consume about 30% of global energy and resource use, significantly contributing to climate change [7]. These challenges are particularly acute in tropical regions, where urban neighborhoods can experience temperatures of 10 to 20 °C higher than nearby rural areas [8]. Such disparities underscore the urgent need for sustainable urban planning and energy-efficient infrastructure.

The increase in temperature due to climate change can affect an individual’s health by increasing the prevalence of heat-related illnesses, respiratory conditions, and stress-induced disorders [9,10]. Therefore, climate change reshapes human experiences by effecting physical and mental health [11]. It can disrupt ecosystems and significantly affect socio-economic structures [12]. Climate change disproportionately impacts populations directly exposed to environmental hazards and those living in vulnerable geographical regions. These groups often face additional challenges due to limited access to resources, information, and protective measures, such as those aimed at reducing vulnerability to climate change [13].

The manifestation of climate change varies significantly based on geographic location. Some countries are highly affected by climate change and face extreme weather events, while others experience less noticeable changes [14]. The United Arab Emirates (UAE), located in the Torrid Zone, exemplifies the complex interaction between rapid development and environmental vulnerability [15]. As one of the world’s leading per capita energy consumers, the UAE relies heavily on fossil fuels yet demonstrates a solid commitment to sustainability through initiatives like the Green Economy Initiative [16] and Masdar City [17]. UAE has seen fluctuating rainfall patterns and has experienced irregular and unpredictable precipitation in recent years [18,19], making the nation vulnerable to sea-level rise [20]. Average temperatures have also increased, and extreme heatwaves are becoming more common [21], aligning with broader global warming trends [22,23]. Recent events, such as the April 2024 floods, highlight the urgency of understanding people’s perception of climate change in the UAE. Although human activities significantly contribute to climate change, varying levels of awareness and belief in its existence and causes affect mitigation and adaptation efforts [24,25].

Individual and collective adaptation efforts are critical to reducing the harm caused by climate change or taking advantage of the opportunities it might bring [26]. Research suggests that private initiatives can yield significant public benefits and foster community-wide resilience [27]. However, studying how individual adaptation would affect public adaptation and maintaining a sustainable society is essential [28]. Adaptation could be affected by experience of hazards and knowledge of climate change and its risks. Education, age, and cultural norms also affect public perceptions of climate change [29].

Prior research has explored the perception of climate change, employing diverse measures and focusing on distinct objectives. The most studied measures are knowledge and climate awareness, perceived adaptive capacity, experience and risk perception, adaptive behavior, and policy support.

Knowledge and Climate Awareness: Public perception and awareness of climate change have been studied in the literature [30,31,32,33]. Education can shape knowledge of and attitudes towards climate change [30] like perception of and response to health risks [33]. People with higher knowledge possess better knowledge of climate actions [31]; however, higher knowledge does not necessarily lead to better actions unless accompanied by changes in attitudes and behaviors [32]. Lack of expertise and education affects climate reporting, and public perception could spread misinformation on climate change [34]. Previous work explores climate awareness but does not study how knowledge directly translates into adaptation behaviors or policy support. This study focuses on linking knowledge to practical adaptation and policy support.

Perceived Adaptive Capacity: Some researchers have focused on studying people’s beliefs about their ability to take the necessary actions to protect themselves and adapt to climate change. At the psychological level, studies showed that values and beliefs about climate change drive personal norms, which influence behaviors and emotional responses [35]. At the practical level, individuals who perceive climate change as a significant risk and feel they have the efficacy to act are more likely to engage in adaptive behaviors [36]. While previous research discusses psychological determinants of climate action, like self-efficacy and emotions, this study focuses on perceived adaptive capacity and actual behavior.

Experience and Risk Perception: The role of experience, especially extreme weather events, and risk perception has been studied previously [33,37,38]. Environmental risks are perceived differently based on age and educational experiences [37]. Individuals who have experienced extreme weather events are more likely to perceive climate risks as urgent and take action [38]. The perception of environmental risks affects the perception of and response to health risks [33]. Previous research has focused on how experience would drive risk perception and concern. However, this study links experience with extreme weather events with adaptive behaviors and policy support.

Adaptation Behavior: Previous research examines how psychological factors and knowledge shape climate adaptation behavior [34,36]. Knowledge about climate change can change people’s beliefs and adaptation behavior. These studies focus on individual and collective action, but adaptation behavior remains understudied at the intersection of policy implementation and perceived capacity.

Policy Support: Climate change policies and public support have been studied in the literature, showing that individuals’ environmental values and their trust in government influence their support for policy measures [39,40]. These studies examine policy acceptance but do not relate it with previous experience with extreme weather events. This study explores the link between experience with extreme weather events and policy support.

Previous work has focused on countries like Pakistan [34] and France [39]; few have focused on UAE. Given the accelerating effects of climate change and the rising frequency of climate-related risks and hazards, understanding public perception and adaptive capacity is critical. This paper studies how experience with extreme weather events, climate change risk perception, and perceived adaptive capacity affect protective measures and individual support for policies in UAE. Table 1 presents a summary of the literature and how it relates to this study. This study employs statistical methods, including descriptive statistics, reliability analysis, and multiple regression, to examine the key factors influencing climate policy support. This paper seeks to bridge gaps in knowledge by exploring the interplay between individual perceptions and collective action, mainly focusing on how natural disasters and policy frameworks influence public attitudes and behaviors.

The remainder of this paper is organized as follows: Section 2 presents the inter-relationship between the variables reviewed, Section 3 describes the research materials and methods, Section 4 presents the results, Section 5 discusses the findings with recommendations and implications for policy and practice, and Section 6 is the conclusion.

## 2. Inter-Relationships Between the Variables Reviewed

Previous work in the literature has studied different variables related to climate change perception. These variables are discussed from different perspectives and found to be interconnected and influence each other. Multiple studies have focused on understanding the connections between variables, such as how climate anxiety, perception of climate change, and behavioral engagement interact [37]. Climate anxiety is a response to the perception of climate change, especially among youth, and often leads to emotional distress [37]. Other variables like knowledge, concern, and policy support connections have been studied [39]. High concern about climate change was linked to experience rather than knowledge, and this concern is positively related to support for climate policies [39]. Similarly, the climate concern was positively related to personal responsibility and engagement in climate actions [35]. High-risk perception could be linked to experience with extreme weather events and environmental sensitivity, which can drive more significant concern about climate change and policy action [38]. Similarly, this high-risk perception and strong efficacy beliefs also positively correlate with climate change adaptation measures [36]. Additionally, the engagement of professionals in climate change is essential. Similarly, more knowledge of climate change leads to a better perception of the issue, which is further influenced by the education level of individuals [30]. People often underestimate global support for climate action, impacting their willingness to advocate for or engage in it [40]. Misinformation in climate change journalism can influence the public’s perception of climate change, potentially leading to misguided actions or lack of action [34]. Climate change awareness is highly correlated to public knowledge of climate change, as moderate awareness can be attributed to gaps in knowledge [32]. There is a positive relationship between public awareness and knowledge about climate change, with education playing a key role in enhancing both [31]. The perception of climate change is strongly tied to health impacts in vulnerable populations, indicating that health concerns may drive public engagement with climate change issues [33]. Based on the relationships identified in the study, the present work aims to investigate how public perception of climate change evolves, especially in response to key events like natural disasters. This study would help understand how behaviors like protective measures and policy support change with increasing experience, risk perception, and perceived adaptive capacity.

## 3. Materials and Methods

The questionnaire used in this study was conducted in the UAE to explore residents’ perceptions of climate change. It examined how their personal experiences with climate change, risk perceptions, and perceived adaptive capacity influence their adaptation behaviors and support for climate-related policies. This section provides a comprehensive overview of the methodology employed in the study.

### 3.1. Questionnaire Design

The questionnaire is structured into five sections, each focused on assessing a specific measure through targeted questions. These measures include risk perception, experience with extreme weather events, perceived adaptive capacity, policy support, and adaptation behavior. All questions are presented on a 5-point Likert scale, ranging from strongly disagree (1) to strongly agree (5), to capture the intensity of participants’ responses. Additionally, the questionnaire incorporates a demographic section, collecting data on gender, age, education level, employment status, marital status, and urban residency. To enhance the analysis of responses, a key question is included to determine whether respondents have previously participated in climate change awareness courses, providing valuable context for their perspectives and experiences.

While Likert-scale responses are technically ordinal, research suggests that when using a 5-point scale or higher, treating them as interval data is statistically appropriate and widely accepted in behavioral and social science research [41,42]. The use of means and standard deviations for descriptive analysis, as well as multiple regression for examining relationships between key variables, aligns with this approach. Sullivan and Artino [43] further explain that parametric tests, including regression, can be applied to Likert-type data when the sample size is sufficiently large and the data distribution approximates normality. Based on these recommendations, this study employs multiple regression analysis to identify significant predictors of policy support, ensuring that the statistical methods used are both valid and widely practiced.

The study assessed five key variables: Risk Perception, Flood Experience, Perceived Adaptive Capacity, Policy Support, and Adaptation Behavior. Each variable was measured using multi-item Likert-scale questions, adapted from established research in climate perception studies.

Risk Perception: Defined as individuals’ awareness and concern regarding climate change risks, this variable was measured using a 5-item Likert scale (1 = Strongly Disagree, 5 = Strongly Agree), adapted from Diakakis et al. [38]. The items assessed perceived risks to health, family, and environmental conditions.

Flood Experience: This variable captures participants’ direct exposure to flooding events. It was measured using five questions evaluating personal and community-level flood impacts. The scale was adapted from Kayode et al. [44].

Perceived Adaptive Capacity: This refers to individuals’ belief in their ability to respond effectively to climate change. It was assessed through three Likert-scale items, based on Acquah [31].

Policy Support: Defined as participants’ willingness to endorse climate-related policies, this variable was measured using four Likert-scale questions, designed to assess support for climate literacy, institutional funding, and governmental initiatives. These items were developed to align with the UAE’s environmental and sustainability strategies.

Adaptation Behavior: This measures proactive climate-related actions taken by individuals, including flood preparedness and community engagement. It was assessed through four Likert-scale questions, referencing previous behavioral adaptation studies.

The questionnaire assessed participants’ experiences with floods due to climate change, explicitly examining UAE residents’ encounters during the April 2024 floods. The questions were meticulously designed, drawing from well-established resources to ensure validity and alignment with the study’s objectives. The risk perception questions were adapted from Diakakis et al. [38], providing insights into individuals’ awareness and concerns about climate change risks. The assessment included five questions designed to evaluate the participant’s general risk perception and perceptions of risk related to health, family, and community. This measure studies the interplay between human beings’ mental awareness and health, physical, and environmental impacts. The flooding experience items were informed by Kayode et al. [44], capturing the direct impacts of the April 2024 events on the participants. This measure also includes five questions to capture the participant’s experience of floods at a personal level, family, and community. The questionnaire utilized methodologies from Acquah [31] to evaluate perceived adaptive capacity. This measure focuses on participants’ beliefs about their ability to prepare for and respond to climate change influenced by knowledge. These three measures define Group A, where questions are carefully structured to examine how these measures influence policy support and protective measures. Table 2 presents the questions proposed for each measure in Group A.

Questions about protective measures were outlined to examine the actions taken to reduce risks and adapt to future flooding challenges. Finally, policy support questions were specifically designed to meet the study’s unique requirements, ensuring relevance to the UAE’s socio-political and environmental context. These two measures constitute Group B, where individuals’ behaviors are assessed. These two measures provide a framework for examining the impact of human behavior and mental acceptance of policies on environmental sustainability and public health outcomes. Table 3 presents the questions proposed for each measure in group B. This approach allowed for a comprehensive evaluation of participants’ flooding experiences and adaptive responses.

### 3.2. Questionnaire Survey

The questionnaire was designed as a Google Form and made publicly accessible via a shared link during the stage of data collection. It was also distributed through professionals to maximize its reach, facilitate its dissemination, and ensure participant responses. A total of 291 participants successfully completed the questionnaire. The sample size was determined based on practical data collection constraints rather than predefined criteria. However, a typical sample size for a 95% confidence level with a 5% margin of error is approximately 384. The sample resulted in a slightly adjusted margin of error, ranging from 0.5 to 0.57, which remains within an acceptable range for social research studies. Additionally, it was ensured that the sample was relevant to the study by excluding respondents from outside the UAE. The survey was conducted in November 2024, targeting individuals above 18 years of age.

### 3.3. Data Analysis

The study began by conducting a statistical analysis to validate the reliability of the survey questions. Reliability was confirmed by analyzing internal consistency, often benchmarked by Cronbach’s alpha, ensuring it exceeded an acceptable threshold, such as 0.6 [45]. Data were analyzed using descriptive statistics for summary insights, Cronbach’s alpha for reliability assessment, and multiple regression analysis to determine significant predictors of policy support. Descriptive statistics, including mean and standard deviation, were computed for each survey item to gain insights into respondents’ perceptions. Statistics for demographic questions were also computed in order to understand how perceptions varied across different demographic groups, such as differences in age categories, enhancing the depth of the analysis. Then, inter-relationships among key constructs, including risk perception, personal experience with flooding, perceived adaptive capacity, protective measures, and support for policy measures, were studied. The analysis primarily sought to uncover the correlation between risk perception and protective measures, exploring how individuals’ awareness of climate-related risks shapes their proactive actions toward risk reduction. Furthermore, the study aimed to assess the role of flood experience in influencing protective measures, delving into how personal encounters with climate events can drive behavioral responses.

## 4. Results

Table 2 provides a statistical analysis of Group A questions related to climate change perception, including experiences, risk perception, and perceived adaptive capacity. The table outlines the percentage of participants responding to each item on the 5-point Likert scale, the mean, and standard deviation for each question. The findings reveal that a substantial proportion of participants selected neutral responses, with the average mean scores between 1.78 and 4.28. Most mean scores exceed 2.7, indicating that participants predominantly responded with neutral, agree, or disagree options rather than SD or SA. An exception is question A5, which highlights that the participants largely agreed there was no loss of lives during the flood. The standard deviation for the questions is close to 1, with the highest standard deviation reported at 1.36. The reliability of the questionnaire is affirmed by Cronbach’s alpha greater than 0.6 for all three measures, experience, risk perception, and perceived adaptive capacity, demonstrating the validity and consistency of the questions. The results further indicate that most participants exhibit a heightened perception of climate change risks, substantial firsthand experience with the recent flood, and a strong perceived adaptive capacity. These insights underscore the participants’ awareness of climate-related challenges and readiness to adapt to potential impacts.

Table 3 presents a detailed statistical analysis of the measures in Group B, which focuses on protective measures related to climate change. The table includes the percentage distribution of participants’ responses across each item on the 5-point Likert scale, the mean, standard deviation, and reliability score. The responses for protective measures show a wide distribution across the Likert scale, with a mean consistently greater than 2.6. This indicates that most participants exhibit agreement or active participation in protective measures. The standard deviation for this measure ranges between 1.30 and 1.40, indicating moderate variability in responses. This variability suggests that while many participants are aligned in their agreement or participation, there is still some divergence in the levels of commitment or frequency of these behaviors. The protective measures measure demonstrates a Cronbach’s alpha of 0.7997, establishing its reliability and validity. The policy support measure reveals that participants’ responses are concentrated in the “SA” category, consistently reflecting the highest percentage across all questions. This trend suggests widespread support for climate-related policies among the participants. The mean values for the policy support questions reinforce this finding, with the lowest mean reported for question B6 at 3.93. This indicates that most participants either agree or strongly agree with the proposed policies. The standard deviation for all questions is approximately 1, signifying relatively low response variability. This consistency implies a consensus among participants regarding the importance and acceptance of climate-related policies. The Cronbach’s alpha of 0.9115 shows that this measure is valid and reliable.

Table 4 provides a statistical summary of the demographic data collected from participants. The results indicate that most respondents were female, unemployed, enrolled in a Bachelor’s degree program, single, and aged between 18 and 25. The high unemployment rate is largely attributed to the significant proportion of participants pursuing their Bachelor’s degrees. Additionally, the analysis reveals that most respondents were from Abu Dhabi, representing 82.13% of the total participants. This demographic distribution highlights the characteristics of the sample population, offering valuable context for interpreting the study’s findings on climate change perceptions and behaviors.

**Figure 1:**
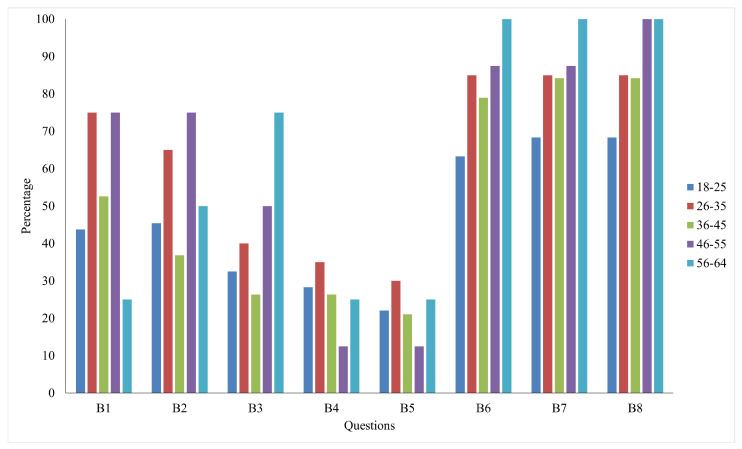
Percentage of distribution of responses for participants across age groups.

Figure 1 shows the percentage distribution of responses that agree with the policy support question and regularly engage in protective measures across five age groups (18–25, 26–35, 36–45, 46–55, and 56–64) for questions categorized into two measures: protective measures (B1 to B5) and policy support (B6 to B8). The 26–35 and 46–55 age groups show the highest engagement percentage in these protective measures, B1 and B2. The young age group (18–25) and old age group (56–64) exhibit comparatively lower engagement. In protective measures, B3 participation drops significantly for all age groups except for the 56–64 group, who reported the highest percentage in B3. This suggests that these behaviors might be more challenging or less prioritized. The age group 26–35 also reported the highest percentage of protective measures, B4 and B5. The two age groups, 18–25 and 36–45, have less participation than others. In policy support, all groups have reported a high percentage of support above 60%, with the highest always reported by age group 56–64.

**Figure 2:**
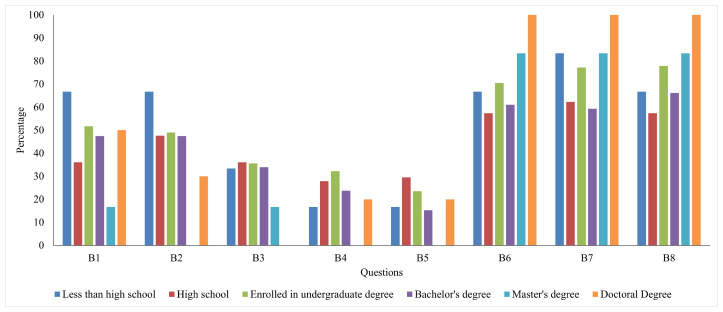
Percentage of distribution of responses for participants across different educational levels.

Figure 2 shows the percentage of responses that agreed with climate change policies and performed different protective measures across different education levels. Respondents with lower educational attainment (“Less than High School”) show the highest engagement in protective measures (B1 and B2), followed by individuals enrolled in a Bachelor’s degree. Interestingly, Master’s degree holders tend to report lower engagement than the “Less than High School” group in these behaviors. The protective measures (B3, B4, and B5) engagement levels drop significantly for all educational levels, with relatively slight variation across the groups. However, respondents with a Bachelor’s degree and High school education show slightly higher engagement than those holding a Master’s degree and Doctoral degree. Support for policy measures is highest among respondents with a Doctoral degree, showing near-universal agreement (close to 100%). Master’s degree holders follow closely, showing similarly high levels of policy support. Individuals with “Less than High School” education still demonstrate strong support but lag behind the more highly educated groups.

**Figure 3:**
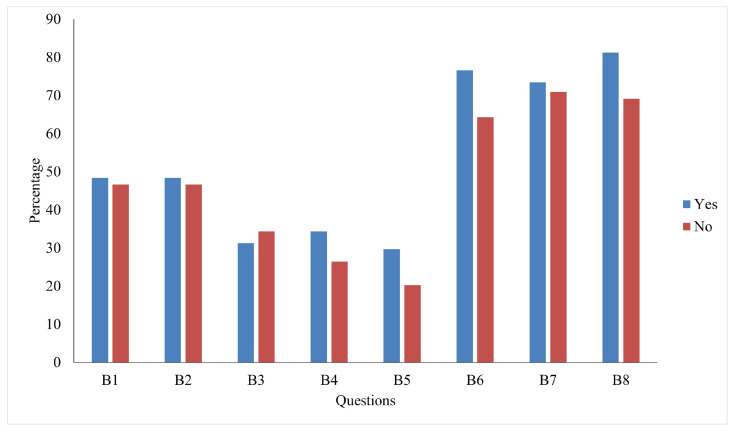
Percentage of distribution of responses for participants who have taken or not taken courses about climate change.

Figure 3 displays a bar chart illustrating the percentage of individuals who agree with the policy support questions and regularly engage in protective measures. The data are divided into two groups: those who have taken an environmental course or possess environmental knowledge (labeled as “Yes”) and those who have not (labeled as “No”). The chart reveals that individuals with prior exposure to environmental education are generally more likely to perform regular protective measures than those without such exposure, except for behavior B3. Although those with higher knowledge tend to perform protective measures, the percentage of participants who perform these behaviors is less than 50%. Similarly, regarding policy support, individuals with prior environmental knowledge show a greater tendency to endorse policies addressing climate change.

To explore the relationship between demographic variables and climate-related measures, a Spearman correlation analysis was conducted. The analysis examined the associations between age, gender, education, and employment status with five key measures: experience with climate events, risk perception, perceived adaptive capacity, protective measures, and policy support. The results revealed notable trends in how different demographic groups engage with climate change. Age exhibited a positive correlation with policy support and adaptive capacity, indicating that older individuals are more likely to support climate policies and feel capable of adapting to climate-related changes. Additionally, gender showed a significant negative correlation with risk perception and policy support, suggesting that women perceive climate risks more seriously and are more supportive of climate-related policies compared to men. Conversely, education and employment status did not display strong correlations with any of the climate-related measures, implying that formal education and professional background may not significantly shape climate perceptions or adaptive behaviors. The following tables summarize the detailed correlation coefficients for each measure. Table 5 summarizes the key findings from the Spearman correlation analysis, highlighting statistically significant relationships (*p* < 0.05) between demographic variables and climate-related measures.

## 5. Discussion

### 5.1. Results Implications

The results of the questionnaire indicate that most participants responded to the survey questions with neutral, agree, or strongly agree, with some disagreeing, and rarely did participants respond with strongly disagree. Participants demonstrated high levels of risk knowledge, perceived adaptive capacity, and personal experience with flooding. Additionally, some participants had previously taken climate change-related courses. Most respondents supported climate change policies, indicating their willingness to accept proposed measures to control climate change. This shows that the individuals with high awareness and experience of floods support integrating climate policies to foster greater knowledge for different groups, thus protecting the environment and health. However, these factors do not significantly influence their engagement in protective measures despite individuals’ high knowledge, education levels, or age. Even among individuals who have experienced extreme weather events and reported property losses, such experiences have not noticeably altered their behavior. This suggests that additional barriers, such as convenience, cost, social norms, or lack of clear incentives, can impede individual action. This behavior could affect public adaptation and hinder the collective effort required to maintain a sustainable society and protect public health. Public adaptation measures may lack the necessary support and effectiveness without widespread individual participation, potentially compromising long-term sustainability goals.

Similarly, participants who have completed environmental courses or received climate change education show a low level of engagement in protective measures. Interestingly, individuals with advanced education levels, such as Master’s or Doctoral degrees, also exhibit low participation in protective measures. This trend is especially pronounced among Master’s degree holders, who reported no engagement in specific behaviors such as B2, B4, and B5. This highlights a disconnect between knowledge, education, and actual behavioral change, suggesting the need for more effective strategies to translate awareness and education into tangible climate action. However, the younger age group, 26–35, participates more in protective measures. The responses to protective measures across different groups of participants suggest that, despite their experience and high level of knowledge, these factors may not significantly influence their actions. This could be attributed to a perception that climate change does not pose long-term effects or a belief that such events have already concluded, reducing the sense of urgency to adopt proactive protective measures.

Understanding how people perceive climate change is critical for managing current and future risks, as it directly influences the development of effective strategies, policies, and interventions. Tailoring communication strategies to address public perceptions can ensure messages resonate with diverse audiences. For instance, emphasizing local and immediate climate-related risks, such as flooding, can be more effective in motivating those who may perceive broader climate change as a distant or abstract issue. Insights into perceptions also enable targeted interventions to address specific behaviors or attitudes that hinder climate action, such as implementing educational programs or incentives to encourage engagement in protective efforts. Additionally, understanding public perceptions supports the design of widely accepted policies, as people are more likely to back initiatives when they recognize the risks of inaction and the benefits of protective strategies like renewable energy investments or conservation measures. From a risk management perspective, aligning strategies with public concerns ensures that efforts are more effective and relevant to communities. This includes prioritizing risk reduction actions, such as infrastructure improvements in areas vulnerable to flooding, while also considering adaptation measures where necessary. Addressing misconceptions and fostering long-term public engagement are essential for sustaining behavioral changes and policy adherence, which is critical for managing future climate risks. Integrating behavioral insights into policy frameworks helps bridge the gap between knowledge and action, ensuring a collective and sustainable approach to combating climate change and avoiding its consequences on health.

The Spearman correlation and regression analyses revealed several key insights into how demographic factors influence climate-related perceptions, adaptive capacity, and policy support.

The analysis revealed that age is positively correlated with flood-related experiences, indicating that older individuals are more likely to have encountered flood damage, disruptions, and losses. In contrast, education and employment status showed weak or no significant correlations with flood-related experiences, suggesting that exposure to climate-related events is not influenced by socio-economic factors. These findings imply that older individuals may be more engaged in climate adaptation strategies, as their past experiences may have heightened their awareness and preparedness. Policymakers can leverage this by involving older individuals in community-driven climate resilience initiatives, where their firsthand knowledge can contribute to local adaptation strategies. Furthermore, the lack of correlation between education, employment, and flood experiences suggests that climate impacts are widespread, affecting all societal groups regardless of their academic or professional backgrounds. This highlights the need for broad and inclusive climate resilience policies that address the general population rather than targeting specific demographic segments.

The findings indicate that age is positively correlated with risk perception, suggesting that older individuals are more likely to view climate change as a significant threat to their health, finances, and environment. Interestingly, higher education does not significantly increase risk perception, challenging the assumption that formal education alone enhances climate awareness. Additionally, employment status has a weak negative correlation, implying that working individuals may not perceive climate risks as strongly, potentially due to prioritizing short-term economic concerns over long-term environmental impacts. These results suggest that older individuals may be more conscious of climate risks due to their life experiences and past exposure to extreme weather events, making them valuable stakeholders in climate adaptation strategies. Moreover, the lack of a strong link between education and risk perception indicates that theoretical knowledge alone is insufficient in fostering climate awareness. Instead, policymakers and educators should focus on experiential learning, real-world engagement, and awareness campaigns that translate climate knowledge into actionable understanding. Additionally, targeted initiatives for working individuals may be necessary to bridge the gap between economic priorities and climate risk perception, ensuring that climate preparedness becomes a fundamental part of workplace policies and decision-making.

The analysis indicates that age has a weak but positive correlation with perceived adaptive capacity, suggesting that older individuals feel slightly more capable of adapting to climate change. However, this correlation is not strong enough to imply full preparedness. Additionally, higher education does not significantly influence adaptive capacity, highlighting that formal education alone may not effectively translate into climate adaptation actions. Similarly, employment status does not strongly impact perceived adaptive capacity, indicating that workplace experience does not necessarily equip individuals with the skills needed for climate adaptation. These findings suggest that while older populations may have developed coping mechanisms over time, additional efforts are needed to strengthen their preparedness. Moreover, the lack of correlation between education and adaptive capacity underscores the need for practical, hands-on climate response training, as theoretical knowledge alone may not be sufficient. To enhance climate resilience, education systems and workplace training programs should integrate applied learning experiences, ensuring that individuals gain the necessary skills to effectively respond to climate challenges.

The findings reveal that age has a moderate positive correlation with protective measures, suggesting that older individuals are more likely to take proactive actions such as securing electrical wiring, clearing drainage, and preparing for floods. In contrast, education does not show a strong correlation with protective behaviors, reinforcing the idea that experience plays a greater role than formal education in shaping climate preparedness. Additionally, gender differences suggest that men and women engage in protective measures differently, which could be attributed to cultural roles and household responsibilities. These results imply that older individuals are more inclined to adopt protective behaviors due to their firsthand experiences with climate-related disasters, making them valuable resources for community resilience efforts. The lack of a strong link between education and protective actions suggests that theoretical knowledge alone does not necessarily translate into practical implementation. This highlights the need for hands-on training and awareness programs that focus on real-world applications, ensuring that climate preparedness strategies are effectively integrated into daily routines across all demographics.

The results indicate that age has a positive correlation with support for climate policies, particularly in areas such as climate education, research funding, and climate literacy. This suggests that older individuals are more likely to advocate for climate-related policies, making them a crucial demographic for climate advocacy efforts. Interestingly, higher education does not significantly increase policy support, indicating that belief in climate policies is not necessarily tied to academic background. Additionally, employment status shows weak or no correlation, suggesting that job roles do not have a strong influence on climate policy preferences. These findings highlight the importance of engaging individuals in climate policy discussions beyond formal education settings, as academic knowledge alone does not automatically translate into policy support. To enhance public endorsement of climate initiatives, policymakers should focus on practical, real-world engagement strategies that connect individuals to the tangible impacts of climate policies, ensuring that advocacy efforts reach diverse societal groups.

### 5.2. Existing and Proposed Policies

The UAE’s primary policy framework, the National Climate Change Plan 2017–2050, is built on a comprehensive structure that integrates best practices and scientific evaluations [46]. It focuses on greenhouse gas mitigation, climate adaptation, and economic diversification through green investments and innovation [46]. The Long-Term Strategy, which details sector-specific measures across power, industry, transport, and buildings, further reinforces the nation’s commitment to sustainable development [47]. In addition, the UAE has shown a strong commitment to climate education, embedding sustainability topics into school curricula and launching initiatives such as the Greening Education Partnership Roadmap in collaboration with UNICEF and UNESCO [48,49]. This roadmap aims to green 50% UAE schools, train thousands of educators in climate literacy, and integrate sustainability in all 23 national curricula [50,51]. Complementary programs, such as the Greening Capacities Initiative and the Educators Training—Climate Education Pioneers Program, further empower youth to participate in environmental decision-making.

Despite these comprehensive efforts, targeted policy enhancements can further strengthen climate resilience and public engagement. Based on the findings of this study, the four key policy recommendations that emerged can complement and expand existing measures:

1. Behavioral Interventions: Although the UAE has introduced policies such as the Green Visa and electric vehicle incentives, expanding climate action through social-norm-driven campaigns could foster sustained public engagement. Research suggests that behaviorally informed strategies can significantly increase pro-environmental behaviors. Integrating these methods into existing programs can enhance their long-term impact.
2. Hands-On Climate Education: The Greening Education Partnership Roadmap aligns well with experiential learning approaches. However, expanding interactive, project-based learning could further bridge the gap between climate knowledge and action. Collaborations with private-sector innovators, NGOs, and local sustainability hubs can provide real-world exposure, ensuring that students understand climate challenges and engage in practical solutions.
3. Localized Climate Policies: While the UAE has made significant progress in renewable energy deployment, ensuring equitable access across all economic segments remains crucial. Further region-specific adaptation strategies can enhance the resilience of local populations. Policies tailored to specific geographical and socio-economic conditions will maximize climate adaptation benefits.
4. Targeted Climate Messaging: The UAE’s public awareness campaigns, including those launched during COP28, have successfully engaged diverse audiences. However, further segmentation of communication strategies could enhance effectiveness. For example, climate messaging tailored by age group, profession, and socio-economic status can increase relevance and engagement. Leveraging social media influencers, educational platforms, and community leaders can improve outreach and encourage broader participation in climate initiatives.

While this study acknowledges the limitations of a smaller sample size, the diversity of participants across different genders, age groups, and educational backgrounds provides meaningful insights. Research indicates that even smaller but demographically varied samples can yield robust qualitative findings [52]. Despite challenges in recruitment, the perspectives gathered reflect key trends in public attitudes toward climate policies, reinforcing the need for these targeted interventions.

The statistical analysis reveals that experience with extreme weather events and climate risks is more strongly associated with adaptive behaviors and policy support than formal education. Contrary to expectations, individuals with higher educational levels or prior exposure to climate-related courses do not consistently engage in protective measures or support climate policies. Instead, age emerged as a significant factor, with older individuals demonstrating greater adaptive capacity, higher risk perception, and stronger climate policy support. Additionally, gender differences were observed, with women showing greater concern for climate risks and stronger support for climate policies than men. These findings underscore the need for climate engagement strategies that go beyond knowledge dissemination, focusing on practical, hands-on experiences and behavioral interventions to encourage protective actions. By shifting from purely educational approaches to real-world application and community-driven initiatives, policymakers can foster greater public participation in climate adaptation efforts, ultimately enhancing environmental protection and safeguarding public health from climate change-related risks.

Based on the findings, several targeted policies are proposed to enhance climate resilience, risk awareness, adaptive capacity, and policy support in the UAE. These policies focus on employing experience, increasing public engagement, and integrating practical adaptation strategies into daily life.

- Benefiting from Older Individuals’ Experiences for Climate Resilience Initiatives: The analysis highlights that older individuals exhibit greater adaptive capacity, risk perception, and support for climate policies, making them a valuable asset in climate resilience initiatives. Their firsthand experience with extreme weather events provides critical insights into disaster preparedness and adaptation strategies. To harness this potential, governments and community organizations should actively involve older individuals in climate response planning, such as leading local resilience networks, participating in flood preparedness programs, and mentoring younger generations on adaptation strategies. Their lived experiences can serve as a practical learning tool for younger populations, bridging the gap between climate knowledge and real-world action. Additionally, policy frameworks should recognize older individuals as key stakeholders in climate decision-making, ensuring that their experiences shape future resilience strategies.
- Prioritizing Workplace and Community-Based Engagement Over Just Academic Education: The findings suggest that formal education does not strongly correlate with risk perception, adaptive capacity, or policy support, indicating that theoretical knowledge alone is insufficient to drive climate action. Instead, workplaces and community settings offer more effective environments for climate engagement by directly linking climate risks to people’s daily lives. Organizations can integrate climate awareness programs into employee training, ensuring that professionals in vulnerable industries—such as construction, tourism, and agriculture—are equipped with practical climate resilience skills. Similarly, community-led initiatives—such as disaster preparedness groups, local sustainability projects, and neighborhood resilience teams—can provide people with hands-on experience in implementing climate adaptation measures. By shifting the focus from classroom-based education to real-world applications, climate action can become more relevant and accessible to diverse populations.
- Offering Incentives and Practical Training to Increase Adaptive Actions: The study underscores that experience, rather than education, plays a key role in climate adaptation, emphasizing the importance of incentivizing practical engagement. Governments and businesses should introduce financial incentives, such as tax reductions, subsidies, or grants, for households and businesses adopting climate adaptation measures. This could include installing flood barriers, using renewable energy sources, or implementing water conservation systems. Additionally, hands-on training programs should be widely available, focusing on climate risk management, disaster preparedness, and sustainable living practices. These initiatives should be tailored to different demographics, ensuring that both younger and older generations have the necessary skills to respond to climate-related challenges. By removing financial barriers and providing practical tools, policymakers can encourage broader participation in climate adaptation efforts.
- Making Climate Education Action-Oriented, Not Just Theoretical: The results indicate that education alone does not significantly drive climate adaptation behaviors or policy support, reinforcing the need for action-oriented learning approaches. Traditional climate education often focuses on awareness and theory but lacks practical implementation strategies. To address this gap, schools and universities should integrate project-based learning, fieldwork, and simulation exercises into climate-related courses. For example, students could participate in real-world sustainability projects, conduct climate risk assessments in their communities, or engage in resilience-building initiatives. Additionally, partnerships between educational institutions and local governments can create interactive climate labs or training centers where students and professionals collaborate on innovative adaptation solutions. By making climate education experiential and problem-solving-oriented, individuals will be better equipped to translate knowledge into meaningful action.

### 5.3. Limitations and Future Research Directions

- Sample Size and Generalizability
  - The study was conducted on a limited sample size, which, while statistically valid at a 95% confidence level, may not fully capture the diversity of public opinion across all demographic groups.
  - Future studies should aim for larger and more representative samples to enhance generalizability.
- Self-Reported Data and Response Bias
  - The study relies on self-reported questionnaire responses, which may be subject to social desirability bias or misinterpretation of survey questions.
  - Future research could incorporate qualitative interviews or mixed-method approaches to provide deeper validation.

## 6. Conclusions

This research has studied the perception of climate change among people in the UAE. It includes a questionnaire of 21 questions divided into multiple sections, where each section tackles a specific measure related to the climate change issue. The measures studied include personal, family, and community losses due to extreme weather events resulting from climate change, especially floods, the risk perception of climate change and how it would affect health and community, and the perceived adaptive capacity, which includes knowledge and beliefs about actions and causes. This research highlights a significant gap between knowledge, education, and adopting climate protective measures. In addition to threat experience and knowledge evaluations, the questionnaire tackled coping evaluations like protective measures and policy support. The questionnaire was made public, and 291 people responded to it from different locations, educational backgrounds, and ages. The study of demographics in relation to behavior shows that education and age do not affect behavior. Similarly, even those with advanced education, personal experiences with extreme weather events, or climate-related training exhibited low engagement in protective measures. The findings indicate that older individuals show higher policy support, while education level does not significantly influence climate action or policy endorsement. This suggests that increasing awareness or formal education alone is insufficient to drive proactive climate adaptation. Instead, experience and real-world engagement play a more significant role in shaping climate perceptions and behaviors. To effectively promote sustainable behavioral change, policymakers must address psychological, social, and structural barriers that hinder action, ensuring that climate initiatives go beyond knowledge dissemination. The research underscores the need for integrated strategies that align policy frameworks, workplace and community engagement efforts, and hands-on education programs to bridge the gap between climate awareness and meaningful action.

Future research should focus on identifying the psychological and socio-economic factors that hinder or motivate protective measures, such as convenience or social norms, and investigate how experiential or applied educational programs influence long-term behavior changes, as opposed to theoretical or informational approaches.

## Figures and Tables

**Table 1:** Summary of Literature.

Reference	Objective	Methodology	Findings	Relevance to Work
Cornejo et al. [30]	Relationship between climate knowledge and perception in agricultural students	Survey-based quantitative study	Found a positive correlation between knowledge and perception of climate change	Supports education as a factor in climate perception
Andre et al. [40]	Global perceptions and support for climate action	Large-scale survey analysis	Perception gap among individuals, thus creating obstacles to advancing climate action	Misconceptions may influence climate advocacy efforts
Ejaz et al. [34]	Influence of misinformation on climate change journalism in Pakistan	Qualitative analysis	Lack of expertise and education affects climate reporting and public perception	Importance of knowledge about climate change
Lee et al. [37]	Youth perception of climate change	A narrative synthesis of existing studies	Youth perceptions vary based on education, media exposure, and cultural background	Highlights the role of education in shaping climate awareness
Douenne and Fabre [39]	French attitudes on climate policies	Public opinion survey	Limited knowledge but high concerns for climate change after the Yellow Vests crisis	Highlights the influence of socio-political events on climate attitudes
Bouman et al. [35]	Climate worry and action	Survey and behavioral analysis	Climate concern leads to action when linked with personal responsibility	Connects psychological drivers to climate action
van Valkengoed et al. [36]	Risk perception and adaptation to climate change	Risk perception and efficacy survey	Beliefs in efficacy increase the likelihood of climate adaptation	Relevant for risk communication strategies
Diakakis et al. [38]	Public risk perception and experience with extreme weather	Survey-based research in Greece	Experience of disasters increases climate concern	Experience plays a role in shaping climate attitudes.
Acquah [31]	Climate knowledge in Ghana	Logistic regression analysis	Public knowledge varies significantly; education is key	Reinforces the role of education in climate awareness
George et al. [33]	Public perception of climate and health impacts in India	Cross-sectional survey	Climate change is perceived to impact multiple health domains.	Links climate change to health awareness
Abuelgasim and Daiban [32]	Climate change awareness in the UAE	Survey-based study	Moderate awareness but gaps in knowledge	Highlights regional awareness differences

**Table 2:** Questionnaire items for the three measures in Group A.

Measure	Strongly Disagree	Disagree	Neutral	Agree	Strongly Agree	Mean	Standard Deviation	Cronbach’s Alpha
Experience								
A1. The flood damaged the roads in your city and it has caused severe damage?	19.93	21.65	33.68	16.49	8.25	2.71	1.20	0.8221
A2. The flood disrupted the movements in your city such that you were not able to move.	20.62	21.31	23.02	23.02	12.03	2.85	1.32	
A3. The flood caused extensive inundation of the community (e.g., residential areas, public spaces)	17.53	16.49	32.30	21.31	12.37	2.95	1.26	
A4. The flood caused a significant loss of valuable properties (e.g., cars, jewelry, or apartments)	27.83	18.56	21.65	17.53	14.43	2.72	1.41	
A5. The flood resulted in the loss of any family members or loved ones and it has high loss impact on the community	63.57	13.06	11.69	4.81	6.87	1.78	1.23	
Risk Perception								
A6. Climate change will have a noticeably negative impact on my health	12.03	17.52	27.49	22.34	20.62	3.22	1.29	0.878
A7. Climate change will have a noticeably negative impact on my financial situation	20.27	18.90	30.93	18.90	11.00	2.81	1.26	
A8. Climate change will have a noticeably negative impact on the environment in which my family and I live	13.40	16.15	25.43	19.25	25.77	3.28	1.36	
A9. Climate change may lead to changes in weather patterns and extreme weather events	7.22	10.65	18.56	26.80	36.77	3.75	1.25	
A10. Climate change may lead to increased flood frequency	7.56	14.78	21.31	28.86	27.49	3.54	1.25	
Perceived Adaptive Capacity								
A11. I am aware that climate change is a real phenomenon	4.47	5.50	15.12	18.21	56.70	4.17	1.15	0.8863
A12. I believe that climate change is an important topic.	3.09	5.16	12.37	19.24	60.14	4.28	1.06	
A13. Climate change is primarily caused by human activities.	3.78	5.84	26.80	20.62	42.96	3.93	1.13	
A14. Climate change is occurring due to the emission of gases from industrial activities and the burning of fossil fuels.	4.81	6.18	23.37	21.31	44.33	3.94	1.17	
A15. Recycling waste is essential for reducing climate change.	4.81	5.84	19.93	21.65	47.77	4.02	1.16	

**Table 3:** Questionnaire items for measures in Group B.

Measure	Strongly Disagree	Disagree	Neutral	Agree	Strongly Agree	Mean	Standard Deviation	Cronbach’s Alpha
Protective Measures								
B1. I regularly check and remove electrical cables and appliances from sockets during heavy rainfall.	14.78	16.15	21.99	19.93	27.15	3.29	1.40	0.7997
B2. I have taken steps to ensure that my electrical wiring is protected from potential water damage.	11.0	17.18	24.74	23.71	23.37	3.31	1.30	
B3. I encourage my neighbors to take preventive actions, such as elevating electrical appliances, to reduce flood risks.	20.62	17.18	28.52	17.53	16.15	2.91	1.35	
B4. I am knowledgeable about physical steps to take for flood mitigation, such as installing flood barriers.	26.46	20.96	24.40	14.43	13.75	2.68	1.37	
B5. I participate in community clean-up activities that focus on preventing blockages such as clearing blockages in my area	33.68	17.52	26.46	9.97	12.37	2.50	1.37	
Policy Support								
B6. It is important to integrate climate change and sustainability topics into the UAE school curriculum to enhance students’ ability to address climate-related challenges.	4.12	9.62	19.25	22.68	44.33	3.93	1.18	0.9115
B7. It is important to offer grants to universities and research institutions for studying climate change and innovative technologies to advance climate solutions in the UAE.	2.75	6.87	18.90	25.77	45.71	4.05	1.08	
B8. It is important to develop national or regional climate literacy standards to ensure that UAE students gain the necessary knowledge and skills to effectively address climate issues.	2.75	5.84	19.59	26.12	45.70	4.06	1.06	

**Table 4:** Demographic Statistics.

Gender	Number	Percentage	Employment Status	Number	Percentage
Female	228	78.35	Employed	79	27.15
Male	63	21.65	Unemployed	199	68.38
			Self-employed	13	4.47
**Education**			**Age**		
Less than high school	6	2.06	18–25	240	82.47
High school	61	20.96	26–35	20	6.87
Enrolled in Bachelor’s degree	149	51.20	36–45	19	6.53
Bachelor’s degree	59	20.28	46–55	8	2.75
Master’s degree	6	2.06	56–64	4	1.38
Doctoral degree	10	3.44			
**Residency**			**Marital**		
Abu Dhabi	239	82.13	Married	23	7.90
Dubai	46	15.81	Single	167	57.39
Sharjah	2	0.69	Divorced	2	0.69
Fujairah	2	0.69	No Response	99	34.02
Ras Al Khaimah	1	0.34			
Ajman	1	0.34			

**Table 5:** Key findings from the Spearman correlation analysis.

Measure	Demographic Factor	Spearman’s *ρ*	*p*-Value
Policy Support	Age	0.16	0.001
Risk Perception	Gender	−0.11	0.032
Adaptive Capacity	Age	0.16	0.036

## Data Availability

The questionnaire items are presented in this paper.
